# Superiority of MALDI-TOF Mass Spectrometry over Real-Time PCR for SARS-CoV-2 RNA Detection

**DOI:** 10.3390/v13050730

**Published:** 2021-04-22

**Authors:** Magda Rybicka, Ewa Miłosz, Krzysztof Piotr Bielawski

**Affiliations:** 1 Department of Molecular Diagnostics, Intercollegiate Faculty of Biotechnology, University of Gdansk and Medical University of Gdansk, Abrahama 58, 80-307 Gdansk, Poland; krzysztof.bielawski@ug.edu.pl; 2 Laboratory of Molecular Biology, Medical University of Gdansk, Marii Skłodowskiej-Curie 3A, 80-210 Gdansk, Poland; milosz@uck.gda.pl

**Keywords:** SARS-CoV-2, COVID-19, RT-PCR, mass spectrometry, epidemic, pandemic, diagnosis

## Abstract

At present, the RT-PCR test remains the gold standard for early diagnosis of SARS-CoV-2. Nevertheless, there is growing evidence demonstrating that this technique may generate false-negative results. Here, we aimed to compare the new mass spectrometry-based assay MassARRAY^®^ SARS-CoV-2 Panel with the RT-PCR diagnostic test approved for clinical use. The study group consisted of 168 suspected patients with symptoms of a respiratory infection. After simultaneous analysis by RT-PCR and mass spectrometry methods, we obtained discordant results for 17 samples (10.12%). Within fifteen samples officially reported as presumptive positive, 13 were positive according to the MS-based assay. Moreover, four samples reported by the officially approved RT-PCR as negative were positive in at least one MS assay. We have successfully demonstrated superior sensitivity of the MS-based assay in SARS-CoV-2 detection, showing that MALDI-TOF MS seems to be ideal for the detection as well as discrimination of mutations within the viral genome.

## 1. Introduction

The pandemic outbreak of coronavirus disease 2019 (COVID-19) is rapidly spreading all over the world. Since the first confirmed infection caused by the novel coronavirus in December 2019, Severe Acute Respiratory Syndrome Coronavirus-2 (SARS-CoV-2) has so far killed almost three million people and infected more than a hundred million people globally [1].

The main challenge in the current management of severe acute COVID-19 (which is now mainly supportive) is the lack of specific antiviral therapies that would be able to arrest its progression [2]. Although an effective vaccine is now available, there is a lot of time needed to achieve herd immunity. Therefore, testing remains a major defense to stop the coronavirus from spreading. According to the WHO and Chinese Center for Disease Control and Prevention (CDC), real-time PCR (RT-PCR) is considered the ‘gold standard’ method for detection of SARS-CoV-2 infection [3]. Nevertheless, studies have shown that RT-PCR is an error-prone method showing false-negative results rates from respiratory samples for SARS-CoV-2 ranging from 1% to 30% [4]. These diagnostic errors are related to a number of preanalytical and analytical factors, including the source of the sample, delays or inadequate storage conditions before arrival to the laboratory, possible errors of RNA extractions, as well as the different quality and sensitivity of detection kits [5]. Additionally, insufficient viral load, the incubation period of the disease, and the presence of mutations that allow virus to escape from the detection may also lead to the risk of obtaining false-negative results [4]. Therefore, the improvement of the COVID-19 diagnosis is urgently needed for obtaining true negative results, allowing one to exit quarantine and avoid virus transmission and recurrence. In fact, several other methods for SARS-CoV-2 detection, both PCR and non-PCR based, are under development or are waiting to obtain regulatory approval. Nevertheless, most of the methods are accepted for research use only (RUO) or have emergency-use authorization (EUA) by the FDA to qualitatively detect SARS-CoV-2 RNA [6].

Recently, a novel assay combining RT-PCR reaction together with high-throughput mass spectrometry processing on the MassARRAY^®^ System (Agena Bioscience^®^, San Diego, CA, USA) has received the CE-IVD mark in Europe for the qualitative detection of SARS-CoV-2 in upper respiratory specimens. The MassARRAY^®^ SARS-CoV-2 Panel (Agena Bioscience, San Diego, CA, USA) utilizes a four-step process composed of a one-step RT-PCR reaction to reverse transcribe viral RNA into cDNA followed by an amplification of the nucleic acid material, primer extension, and matrix-assisted laser desorption ionization time-of-flight mass spectrometry (MALDI-TOF MS) separation of the products on a matrix-loaded silicon chip array. The MassARRAY^®^ System has been already successfully applied for the detection of high-risk oncogenic variants of human papillomavirus (HPV) [7], drug-resistant variants of hepatitis B virus (HBV) [8], and universal coronavirus screening [9].

The purpose of this study was to evaluate the clinical and diagnostic use of the new commercially available MassARRAY^®^ SARS-CoV-2 Panel from Agena Bioscience. Furthermore, the accuracy of this MALDI-TOF MS-based assay was compared to the RT-PCR diagnostic test approved for clinical use.

## 2. Materials and Methods

### 2.1. Sample Collecton, Storage and Workflow

Oral and nasopharyngeal swabs from suspected patients were collected according to the WHO recommendation at the COVID-19 drive-thru screening center and transferred to the Laboratory of Molecular Biology, Medical University of Gdansk, for the officially approved RT-PCR diagnosis between October and November of 2020. The study group consisted of 168 suspected patients with symptoms of a respiratory infection. Total RNA was extracted by EliGene^®^ Viral DNA/RNA Isolation Kit (Elisabeth pharmacon, BRNO-Zidenice, Czech Republic), GeneProof PathogenFree RNA Isolation Kit (GeneProof, Brno, Czech Republic), or the MagNA Pure LC Total Nucleic Acid Isolation Kit (Roche Diagnostics, Mannheim, Germany). The VIASURE SARS-CoV-2 Real Time PCR Detection Kit targeting ORF1ab and N genes (Certest Biotec, Zaragoza, Spain) was used for SARS-CoV-2 detection. Residual de-identified RNA samples previously diagnosed were stored at −80 °C until further analysis. All participants gave their written consent to participate in this study. Mass spectrometry (MS)-based and RT-PCR assays were conducted simultaneously and immediately after RNA defrosting to avoid differences due to RNA degradation. All procedures were performed on ice. Official test results information was collected after the performance of laboratory tests.

### 2.2. Real–Time PCR

CE-IVD certified Vitassay qPCR SARS-CoV-2 kit (Vitassay Healthcare, Huesca, Spain) was used for the qualitative detection of 2019 Novel Coronavirus by real-time amplification of specific conserved fragments within *ORF1ab* and *N* viral genes. The product provided a SARS-CoV-2-positive and -negative control. The procedure was conducted on LightCycler^®^ 480 II instrument according to the manufacturers’ recommendation. Briefly, 15 µL of resuspention buffer were added to each well containing all the necessary reagents in a stabilized format, and 5 µL of RNA sample was used as an input for the one-step real-time PCR. Positive and negative samples were included at each run. The amplification of the target sequence within *ORF1ab* and *N* genes was detected through the FAM and ROX channels, respectively, whereas the internal control (IC) was detected in HEX channel. The limit of detection (LOD) was ≥10 viral RNA copies per reaction for the ORF1ab and N genes.

### 2.3. Mass Spectrometry-Based Assay

**Figure 1:**
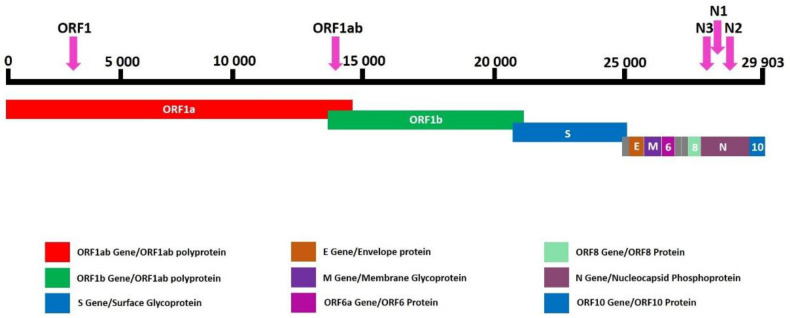
SARS-CoV-2 genome and approximate locations of targets in the MassARRAY SARS-CoV-2 panel.

**Figure 2:**
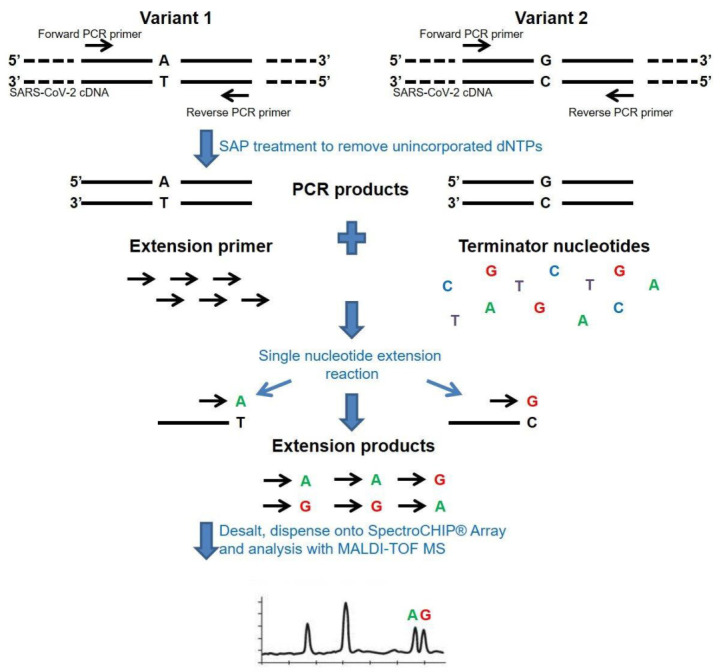
The molecular principle of the mass spectrometry method for cDNA/DNA analysis. The analysis begins with a reverse transcription of viral RNA into cDNA and amplification by PCR in a single reaction. Next, PCR products are treated with an enzyme that removes unincorporated nucleotides (SAP), and a single base primer extension (SBE) reaction is carried out. In the SBE reaction, each product is detected at a specific mass because of the addition of terminating nucleotides, which are complementary to the template sequence.

**Figure 3:**
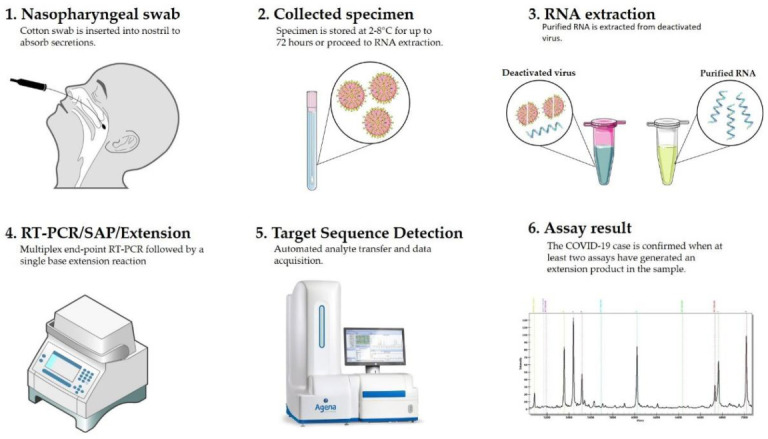
Workflow of COVID-19 diagnostic test through mass spectrometry method.

The CE-IVD marked MassARRAY^®^ SARS-CoV-2 Panel (Agena Bioscience, USA) was used for the detection of SARS-CoV-2 virus on the MassARRAY^®^ System. This multiplexed single reaction allowed for the detection of five SARS-CoV-2 specific targets—three in the N (nucleocapsid) region and two in the ORF1ab region (Figure 1). All assays were designed in the conserved regions of the SARS-CoV-2 genome as available in the National Center for Biotechnology Information (NCBI) database as of 18 August 2020. Three µL of each RNA sample, the positive control and negative control, were used as an input for the one-step reverse transcription of RNA and PCR amplification of cDNA in a final volume of 5 µL (Table 1). Twist Synthetic SARS-CoV-2 RNA Control 1 (Twist Bioscience, San Francisco, CA, USA) was used at each run as a positive control in working stock of 16.7 copies/µL. An additional assay for the MS2 bacteriophage was used an exogenous quality control (QC). After cDNA amplification, unincorporated dNTPs were removed by shrimp alkaline phosphatase treatment. Next, the manufacturers’ standard protocol was followed for iPLEX Pro chemistry (Agena Bioscience, San Diego, CA, USA) and allele-specific products of distinct masses were obtained (Figure 2). The final extension products were diluted with 41 µL ultrapure water and transferred into Chip Prep Module (Agena Bioscience, San Diego, CA, USA) for automated sample handling (Figure 3) including desalting and dispensing samples onto the SpectroChip Array (Agena Bioscience, San Diego, CA, USA). Mass spectra were acquired with a MassARRAY^®^ Analyzer 4 mass spectrometer and analyzed with MassARRAY^®^ Typer 4.0 software. All procedures were performed according to the company recommendations. An estimated LOD of the assay was the genome copy number equivalents (GCE) of the SARS-CoV-2 virus per μL (400 copies/mL).

## 3. Results

A total of 133 samples were reported as positive by the officially approved RT-PCR positive with a relative viral titer range of 10–10^8^ copies/reaction (calculated based on the positive control). Samples were considered positive if both viral genes (*N* gene and *ORF1ab*) and the internal control were detected with the cut-off Ct values of ≤38 and ≤35, respectively. For 15 samples only, one SARS-Co-V2 gene was found (Ct ≤ 38), and due to the internal control presence they were judged as a suspected infection. Twenty samples were reported as negative as no products were found for either: the *N* and the *ORF1ab* genes are negative (Ct > 38) with simultaneous detection of IC. No clinical data were available except patients’ gender (48% males, 52% females).

**Figure 4:**
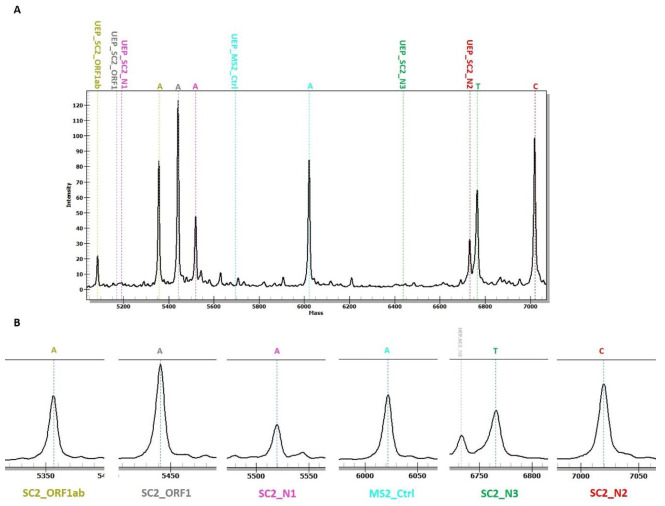
An example of MassARRAY^®^ System data obtained for a positive sample with all SARS-CoV-2 targets detected (**A**). The positions of all extension primers and analyte peaks are labeled with the same color. Mass is in daltons (Da). Zoomed spectra of obtained amplicons are shown in (**B**). UEP—unextended primer.

**Figure 5:**
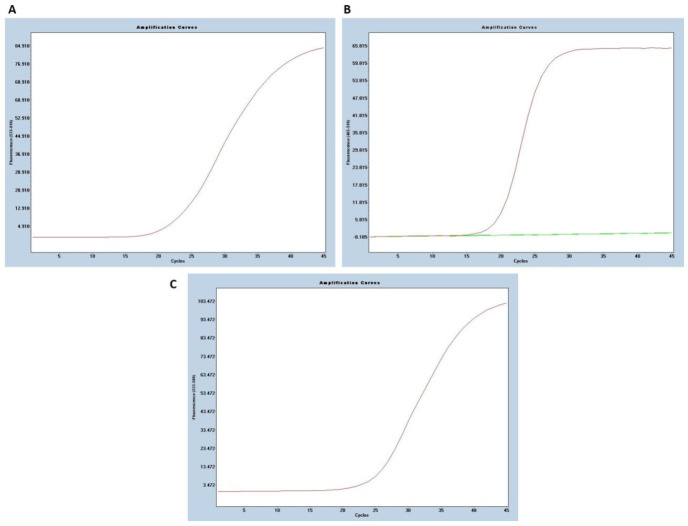
An example of a positive RT-PCR (Vitassay qPCR SARS-CoV-2 kit) result. (**A**) Beta-coronavirus detection in the ROX channel (*N* gene), (**B**) SARS-CoV-2 specific amplification in the FAM channel (*ORF1ab* gene), (**C**) Signal for the RNA control in the HEX channel.

All samples were successfully analyzed and passed the quality control for both RT-PCR and MS-based assays after arrival as double-blinded at our laboratory. There were no differences in SARS-CoV-2 detection rates between RNA extraction methods. Positive results for 133 samples were concordant for both methods (Figure 4 and Figure 5) as well as for the results acquired from the hospital diagnostic laboratory. The MassARRAY^®^ SARS-CoV-2 Panel confirmed the COVID-19 case when at least two or more assays generated an extension product in the sample (Table 2). If just one SARS-CoV-2 target was detected, the samples were qualified as a suspected infection; for the negative result, samples passed the MS2 QC assay, and no viral product was detected. For the Vitassay qPCR SARS-CoV-2 kit, we applied the same cut-off Ct values as were used as in the official RT-PCR test.

Vitassay SARS-CoV-2 positive control and Twist Synthetic SARS-CoV-2 RNA control were analyzed by both RT-PCR and MS-based methods according to the manufacturers’ recommendation. All regions within the SARS-CoV-2 genome were detected when using 3 µL of Vitassay positive control for MassARRAY^®^ SARS-CoV-2 Panel, and both genes were detected when using 5 µL (16.7 copies/µL) of Twist Synthetic SARS-CoV-2 RNA Control 1 for RT-PCR (*ORF1ab* gene: Ct = 35.73, *N* gene: Ct = 37.24). No viral mutants were detected in the study group.

Within fifteen samples previously reported as presumptive positive, a single ORF1ab gene was detected in four, and an N gene in eight samples according to the Vitassay result. The Ct range for *ORF1ab* and *N* gene was 34.72–36.20 and 34.26–37.20, respectively. For the remaining three samples, no viral target was found and they were judged as negative. Thirteen samples had a positive result in at least two of five MS assays. The success rate of viral gene detection was as follows: 85% for *N1*, 77% for *N3*, 69% for *ORF1* and *N2*, and 46% for *ORF1ab*. For two samples, only the *ORF1* gene was detected (Table 3). Moreover, among 20 samples reported by officially approved RT-PCR as negative, all were negative after Vitassay, but four were positive in at least one assay (Table 3).

## 4. Discussion

Population-wide testing for SARS-CoV-2 virus presence was the only way to reduce infection prevalence before the development of the vaccine. Now, when an effective vaccine is available, this strategy is still of importance while substantial proportion of a population remain to be vaccinated in order to achieve herd immunity against COVID-19. Nowadays, the diagnosis of SARS-CoV-2 infection relies on a direct viral RNA detection by RT-PCR assays which target one or more viral genes. Here, we have demonstrated that the MALDI-TOF MS method (MassARRAY^®^ SARS-CoV-2 Panel) is a more accurate tool to detect SARS-CoV-2 virus in comparison to routinely used methods based on RT-PCR. Among the 168 analyzed samples, discordant results were found for 10.12% (17 samples). Notably, in four samples from people with COVID-19 symptoms with two RT-PCR negative results (from both our and the diagnostic laboratory), viral genes were identified by the MS-based method when using the same samples. Two samples were judged as suspected, the other two as positive. Furthermore, 87% (13/15) of patients previously judged as suspected (only one viral gene was detected in the diagnostic laboratory) were clearly positive in all MS assays performed in our laboratory. For the remaining two samples, we found a product in one out of five MS assays and labeled them as suspected. Using the MassARRAY^®^ SARS-CoV-2 Panel, we were able to detect SARS-CoV-2 in low viral load specimens indicating that supposed negative patients are probably carrying SARS-CoV-2, and they should stay in quarantine to avoid the potential risk of severe disease progression as well as viral spreading. Moreover, we have demonstrated that with the use of as little as 3 ul of viral RNA it is possible to confirm the virus’ presence and simultaneously analyze its crucial mutations. Although there were no mutants found in our study, this possibility is now urgently needed as the virus mutates constantly, and more and more new variants appear with potential clinical importance [10,11].

Our data analysis is concordant with other studies reporting false-negative results from RT-PCR tests ranging up to 30% [4]. Several studies have already showed that the diagnostic efficacy between RT-PCR test kits for SARS-CoV-2 nucleic acid detection may differ significantly depending on the probes and primers used [12,13]. Moreover, in a recent systematic review, it was suggested that up to 54% of COVID-19 patients may have an initial negative RT-PCR result [5]. There are several potential sources causing these differences, which include sampling, storage and processing, RNA extraction, cDNA synthesis and amplification, as well as post-analytical steps (interpretation and analysis) [14]. Although similar issues may affect the final result of MS-based assays, our work highlights the advantage of MassARRAY^®^ technology for SARS-CoV-2 diagnosis (Table 4). It is particularly important in patients harboring the virus at low titers, which remains unidentified after an RT-PCR test as they may have the ability to spread the virus to others [15]. Moreover, mass spectrometry technology allows one to provide a very cost-effective analysis as it provides multiplexed assays and eliminates fluorescent markers. In contrast to RT-PCR that uses indirect detection of a product via fluorescent labels, the MS-based method measures the physical property of a target (its mass), eliminating the potential source of doubt and error. Furthermore, the MassARRAY^®^ System combines automation, minimal hands-on time, and onboard data analysis, resulting in a simple and fast workflow. Although the time required for the whole procedure is ~8 h, all steps on a 96-titer plate may be performed at any 96-well thermal cycler. Only the final step including sample conditioning and transferring into a CHipArray^®^ takes place in the MassARRAY^®^ Analyzer. Therefore, samples may be prepared for analysis at different laboratories and then transported for the measurement. Lastly, the MassARRAY^®^ System delivers easy-to-interpret data that do not require a complicated training off stuff.

Despite the relatively large sample size, this study has some limitations. First, we did not consider the specimen quality and isolated RNA concentration. Nevertheless, these issues are not taken into account during a routine diagnosis. Second, there were no opportunities to reanalyze suspected patients to estimate whether the infection had progressed with time.

## 5. Conclusions

In conclusion, we have successfully presented the clinical utility of the MassARRAY^®^ System for the detection of the SARS-CoV-2 virus. Moreover, we have demonstrated a superior sensitivity of the MS-based assay in viral RNA detection, showing that MALDI-TOF mass spectrometry seems to be ideal for the detection as well as discrimination of mutations within the SARS-CoV-2 genome.

## Figures and Tables

**Table 1:** Compositions of the reagent mixtures for all steps of the MassARRAY^®^ SARS-CoV-2 Panel.

Reagent	Per Reaction (µL)
RT-PCR	
10×PCR Buffer	0.500
MgCl_2_, 25mM	0.400
dNTP Mix, 25mM	0.100
UNG	0.050
RNase Inhibitor	0.125
PCR Enzyme	0.200
MMLV Enzyme	0.125
SARS-CoV-2 PCR Primers	0.500
Sample RNA	3
SAP Cocktail	
HPLC-grade water	1.53
SAP Buffer	0.17
Shrimp Alkaline Phosphatase (SAP)	0.3
Extension Cocktail	
HPLC-grade water	0.62
iPLEX Buffer Plus, GPR	0.20
iPLEX Termination Mix	0.20
iPLEX Pro Enzyme	0.04
SARS-CoV-2 Extend Primers	0.94

All reagents are included in MassARRAY^®^ SARS-CoV-2 Panel.

**Table 2:** Rules for sample qualification and virus detection status.

SARS-CoV-2 Targets	MS2	QC Status	Judgement
≥2 SARS-CoV-2 target detected	Detected	Passed	Positive
<2 SARS-CoV-2 target detected	Detected	Passed	Suspected
0–5 SARS-CoV-2 target detected	Not detected	Failed	Invalid

MS2—MS2 RNA bacteriophage (internal control); QC—quality control.

**Table 3:** RT-PCR and MassARRAY^®^ System results obtained for 35 samples.

Sample No	Official Report by RT-PCR ^a^	Results of RT-PCR ^b^		Results of MassARRAY^®,c^					MassARRAY^®^ Judgment
		*ORF1ab*	*N*	*ORF1ab*	*ORF1*	*N1*	*N2*	*N3*	
1	S	35.92	ND	ND	P	P	P	P	P
2	S	ND	38.37	ND	P	P	ND	P	P
3	S	ND	ND	ND	P	ND	ND	ND	S
4	S	ND	ND	P	ND	ND	P	ND	P
5	S	ND	36.24	ND	P	P	ND	P	P
6	S	ND	36.85	ND	P	P	P	P	P
7	S	ND	34.26	P	ND	ND	P	ND	P
8	S	ND	37.20	ND	P	P	ND	P	P
9	S	ND	ND	ND	P	ND	ND	ND	S
10	S	ND	35.96	ND	ND	P	P	ND	P
11	S	34.72	ND	P	P	P	ND	P	P
12	S	36.20	ND	P	P	P	P	P	P
13	S	35.44	ND	ND	ND	P	P	P	P
14	S	ND	35.35	P	P	P	P	P	P
15	S	ND	35.48	P	P	P	P	P	P
16	N	ND	ND	ND	ND	ND	ND	ND	N
17	N	ND	ND	ND	P	ND	ND	ND	S
18	N	ND	ND	ND	ND	ND	ND	ND	N
19	N	ND	ND	ND	ND	ND	ND	ND	N
20	N	ND	ND	ND	ND	ND	ND	ND	N
21	N	ND	ND	ND	ND	ND	ND	ND	N
22	N	ND	ND	ND	ND	P	ND	P	P
23	N	ND	ND	ND	ND	ND	ND	ND	N
24	N	ND	ND	ND	P	ND	ND	ND	S
25	N	ND	ND	ND	ND	ND	ND	ND	N
26	N	ND	ND	ND	ND	ND	ND	ND	N
27	N	ND	ND	ND	ND	ND	ND	ND	N
28	N	ND	ND	ND	ND	ND	ND	ND	N
29	N	ND	ND	ND	P	P	ND	P	P
30	N	ND	ND	ND	ND	ND	ND	ND	N
31	N	ND	ND	ND	ND	ND	ND	ND	N
32	N	ND	ND	ND	ND	ND	ND	ND	N
33	N	ND	ND	ND	ND	ND	ND	ND	N
34	N	ND	ND	ND	ND	ND	ND	ND	N
35	N	ND	ND	ND	ND	ND	ND	ND	N

P, positive; ND, not detected; N, negative; ^a^ VIASURE SARS-CoV-***2*** Real Time PCR Detection Kit (Certest Biotec, Zaragoza, Spain); ^b^ Vitassay qPCR SARS-CoV-2 kit (Vitassay Healthcare, Huesca, Spain); ^c^ MassARRAY^®^ SARS-CoV-2 Panel (Agena Bioscience, San Diego, CA, USA).

**Table 4:** Comparison of both methods for the detection of SARS-CoV-2.

Technique	RT-PCR	MassARRAY^®^ System
Targets	*ORF1ab*, *N*	*ORF1ab, ORF1, N1, N2, N3*
RNA volume	5 µL	3 µL
Time needed *	4–6 h	8 h
Cost	~10 EUR/sample	~10 EUR/sample
Report method	Qualitative	Qualitative
Advantages	Short time of analyses; specific, confirms active cases; useful in clinical decision-making	Highly sensitive, accurate, high-throughput, easy data analysis, cost-effective, simple partially automated workflow, runs up to eight 96-well plates per day, may process two more plates overnight.
Disadvantages	Unable to simultaneously detect virus and its mutations, less accurate in low viral load samples	Multiple preparation steps, requires specialized equipment

* Time needed for the analysis of two 96-well plates.

## Data Availability

All the data supporting reported results can be found at https://mfr.osf.io/render?url=https%3A%2F%2Fosf.io%2Fsv5qt%2Fdownload (accessed on 25 March 2021).

## References

[1] Coronavirus Update (Live): 92,895,303 Cases and 1,989,457 Deaths from COVID-19 Virus Pandemic—Worldometer. https://www.worldometers.info/coronavirus.

[2] Bartoli A., Gabrielli F., Alicandro T., Nascimbeni F., Andreone P. (2021). COVID-19 treatment options: A difficult journey between failed attempts and experimental drugs. Intern. Emerg. Med..

[3] Mukhopadhyay C.D., Sharma P., Sinha K., Rajarshi K. (2021). Recent trends in analytical and digital techniques for the detection of the SARS-Cov-2. Biophys. Chem..

[4] Kanji J.N., Zelyas N., MacDonald C., Pabbaraju K., Khan M.N., Prasad A., Hu J., Diggle M., Berenger B.M., Tipples G. (2021). False negative rate of COVID-19 PCR testing: A discordant testing analysis. Virol. J..

[5] Arevalo-Rodriguez I., Buitrago-Garcia D., Simancas-Racines D., Zambrano-Achig P., Del Campo R., Ciapponi A., Sued O., Martinez-Garcia L., Rutjes A.W., Low N. (2020). False-negative results of initial RT-PCR assays for COVID-19: A systematic review. PLoS ONE.

[6] Carter L.J., Garner L.V., Smoot J.W., Li Y., Zhou Q., Saveson C.J., Sasso J.M., Gregg A.C., Soares D.J., Beskid T.R. (2020). Assay Techniques and Test Development for COVID-19 Diagnosis. ACS Cent. Sci..

[7] Basu P., Chandna P., Bamezai R.N.K., Siddiqi M., Saranath D., Lear A., Ratnam S. (2011). MassARRAY spectrometry is more sensitive than PreTect HPV-Proofer and consensus PCR for type-specific detection of high-risk oncogenic human papillomavirus genotypes in cervical cancer. J. Clin. Microbiol..

[8] Rybicka M., Stalke P., Dreczewski M., Smiatacz T., Bielawski K.P. (2014). High-Throughput Matrix-Assisted Laser Desorption Ionization-Time of Flight Mass Spectrometry as an Alternative Approach to Monitoring Drug Resistance of Hepatitis B Virus. J. Clin. Microbiol..

[9] Xiu L., Zhang C., Wu Z., Peng J. (2017). Establishment and application of a universal coronavirus screening method using MALDI-TOF mass spectrometry. Front. Microbiol..

[10] Zeng L., Li D., Tong W., Shi T., Ning B. (2021). Biochemical features and mutations of key proteins in SARS-CoV-2 and their impacts on RNA therapeutics. Biochem. Pharmacol..

[11] Greaney A.J., Starr T.N., Gilchuk P., Zost S.J., Binshtein E., Loes A.N., Hilton S.K., Huddleston J., Eguia R., Crawford K.H. (2021). Complete Mapping of Mutations to the SARS-CoV-2 Spike Receptor-Binding Domain that Escape Antibody Recognition. Cell Host Microbe.

[12] van Kasteren P.B., van Der Veer B., van den Brink S., Wijsman L., de Jonge J., van den Brandt A., Molenkamp R., Reusken C.B., Meijer A. (2020). Comparison of seven commercial RT-PCR diagnostic kits for COVID-19. J. Clin. Virol..

[13] Lu Y., Li L., Ren S., Liu X., Zhang L., Li W., Yu H. (2020). Comparison of the diagnostic efficacy between two PCR test kits for SARS-CoV-2 nucleic acid detection. J. Clin. Lab. Anal..

[14] Rahbari R., Moradi N., Abdi M. (2021). rRT-PCR for SARS-CoV-2: Analytical considerations. Clin. Chim. Acta.

[15] Zhou R., Li F., Chen F., Liu H., Zheng J., Lei C., Wu X. (2020). Viral dynamics in asymptomatic patients with COVID-19. Int. J. Infect. Dis..

